# Tanyu Tongzhi Formula relieves the progression of atherosclerotic plaque through lipid regulation and anti-inflammatory effects

**DOI:** 10.3389/fcvm.2025.1614525

**Published:** 2025-09-29

**Authors:** Tingting Chen, Lingling Xie, Yan Zhao, Li Wu, Shishi Huang, Wei Mao

**Affiliations:** ^1^ The First School of Clinical Medicine, Zhejiang Chinese Medical University, Hangzhou, Zhejiang, China; ^2^ Zhejiang Key Laboratory of Integrative Chinese and Western Medicine for Diagnosis and Treatment of Circulatory Diseases, Zhejiang Hospital (Affiliated Zhejiang Hospital, Zhejiang University School of Medicine), Hangzhou, Zhejiang, China; ^3^ Zhejiang Engineering Research Center for Precise Diagnosis and Innovative Traditional Chinese Medicine for Cardiovascular Diseases, Zhejiang Hospital (Affiliated Zhejiang Hospital, Zhejiang University School of Medicine), Hangzhou, Zhejiang, China; ^4^ Department of Cardiology, Zhejiang Hospital (Affiliated Zhejiang Hospital, Zhejiang University School of Medicine), Hangzhou, Zhejiang, China

**Keywords:** traditional Chinese medicine formula, Tanyu Tongzhi Formula, atherosclerosis, inflammation, lipid

## Abstract

**Objective:**

Tanyu Tongzhi Formula (TTF), a clinically proven empirical prescription, has been utilized to treat atherosclerosis (AS) for decades. This study aimed to investigate the therapeutic mechanisms of TTF against AS by integrating bioinformatics, multi-omics, and experimental validation.

**Methods:**

The metabolites of TTF in serum were identified using Ultra Performance Liquid Chromatography-Mass Spectrometry (UPLC-MS). Bioinformatics was employed to predict drug targets and mechanisms of action. ApoE^−/−^ C57BL/6J mice were fed a 12-week high-fat diet to establish an AS model and were treated with TTF crude extract (2.25 g/kg/d) via gavage. Interleukin 6 (IL-6) and interleukin 1β (IL-1β) were measured at weeks 6, 10, and 12. At the 12-week endpoint, aortic plaque formation and liver histopathology were evaluated. Liver transcriptomics and serum-targeted lipid metabolomics were performed to assess TTF's regulatory effects on lipid metabolism. *in vitro*, peritoneal macrophages (PMs) were pretreated with TTF-containing serum for 1 h before LPS (2 µg/ml) stimulation. IL-6 and interleukin 10 (IL-10) mRNA were measured by RT-PCR, while NOD-like receptor thermal protein domain associated protein 3 (NLRP3), IL-1β, interleukin 18 (IL-18) and IL-6 expression were assessed by Western blot (WB).

**Results:**

Bioinformatics identified 28 key targets of TTF in AS treatment, primarily associated with inflammation and lipid metabolism. TTF significantly reduced aortic plaque area, attenuated hepatic steatosis, and enhanced plaque collagen content. It decreased the serum levels of lipids and pro-inflammatory mediators (IL-6 and IL-1β) in AS mice. Sphingolipids are the most significantly different lipids. In LPS-stimulated PMs, TTF suppressed IL-6 mRNA and NLRP3 inflammasome activation while upregulating IL-10 mRNA.

**Conclusions:**

TTF exerts its anti-atherosclerotic effect through inflammation reduction. These findings provide a scientific basis for its clinical application in AS treatment.

## Introduction

Atherosclerosis (AS) is a lipid-driven, chronic progressive vascular inflammation characterized by the formation of plaque within the arterial intima. These unstable plaques can lead to arterial lumen constriction, increased impedance to blood flow, a heightened risk of thrombus formation, and even the occurrence of atherosclerotic cardiovascular diseases (ASCVD) (1). With the aging of the population, ASCVD has become a leading cause of global morbidity and mortality, attracting worldwide attention and urgently requiring intervention. Dysregulation of lipid metabolism and inflammatory response are high-risk factors of AS (2).

Traditional Chinese Medicine (TCM) has played a pivotal role in managing complex and chronic conditions such as AS (3). Its multi-compounds, multi-targets and multi-pathways provided new treatment strategies for cardiovascular diseases (CVD) (4). The Tanyu Tongzhi Formula (TTF) is a modified formulation based on the classical TCM prescription “Gualou Xiebai Banxia Decoction”, originally documented in the “Golden Chamber Yao Lve” (5). It has been used to treat AS in our hospital for decades. A real-time cell analyzer was employed to meticulously track the responses of COS-7 cells to TTF, and revealed that TTF aligned with the “emperor, minister, assistant, and envoy” herbal compatibility principle at the cellular level (6).

With the advancement of bioinformatics and multi-omics, the mechanisms underlying the TTF in treating AS are being gradually elucidated. In this study, Ultra Performance Liquid Chromatography-Mass Spectrometry (UPLC-MS) was utilized to identify the metabolites of TTF present in the bloodstream. The SwissTargetPrediction database was employed to predict TTF-related targets, and the TTD, DrugBank, KEGG, and OMIM databases were used to predict AS-related targets. By intersecting TTF-related targets and AS-related targets, we have obtained potential targets for TTF treatment of AS. Gene Ontology (GO) and Kyoto Encyclopedia of Genes and Genomes (KEGG) were conducted to investigate the biological processes (BP) and pathways involved in TTF's therapeutic effects on AS. The results revealed that TTF alleviated AS by modulating inflammation and lipid metabolism. Subsequently, we established an AS model to evaluate the regulatory effects of TTF on pro-inflammatory factors during AS progression. *In vitro*, an LPS-induced macrophage model was employed to further confirm that TTF can suppress inflammatory responses. Our findings provide a theoretical foundation for the application of TTF in AS treatment.

## Materials and methods

### Preparation of TTF crude extract

The TTF was composed of eight drugs: 15 g of *Trichosanthes kirilowii Maxim* [Cucurbitaceae; Trichosanthis Fructus], 15 g of *Salvia miltiorrhiza Bunge* [Lamiaceae; Salviae miltiorrhizae radix et rhizoma], 10 g of *Allium macrostemon Bunge* [Amaryllidaceae; Bulbus Allii Macrostemonis], 5 g of *Leech* [Hirudinidae; Hirudo], 10 g of *Acorus gramineus Aiton* [Araceae; Acori Tatarinowii Rhizoma], 15 g of *Curcuma aromatica Salisb* [Zingiberaceae; Curcumae Radix], 20 g of *Poria cocos* [Polyporaceae; Wolfiporia cocos (F.A. Wolf) Ryvarden and Gilb], and 10 g of *Citrus aurantium L* [Rutaceae; Citri Reticulatae Pericarpium] (Table 1).

**Table 1:** Detailed information on eight drugs in TTF.

Chinese name	Scientific names	Medicinal part	Amount (g)	Place of origin	Authority
Quán guā lóu	Trichosanthes kirilowii Maxim	Seed	15	Shangdong, China	Pharmacopoeia of China (2020)
Dān Shēn	Salvia miltiorrhiza Bunge	Root and Rhizome	15	Anhui, China	Pharmacopoeia of China (2020)
Xiè Bái	Allium macrostemon Bunge	Bulb	10	Shangdong, China	Pharmacopoeia of China (2020)
Shuǐ zhì	Leech	Body	5	Shangdong, China	Pharmacopoeia of China (2020)
Shí Chāng Pú	Acorus gramineus Aiton	Root and Rhizome	10	Shangdong, China	Pharmacopoeia of China (2020)
Yù Jīn	Curcuma aromatica Salisb	Root and Rhizome	15	Zhejiang, China	Pharmacopoeia of China (2020)
Fú Lín	Poria cocos	Sclerotium	20	Zhejiang, China	Pharmacopoeia of China (2020)
Chén Pí	Citrus aurantium L	Peel	10	Guangdong, China	Pharmacopoeia of China (2020)

All crude drugs were authenticated by Prof. Wang Jinxia and Zheng Minxia (The First Affiliated Hospital of Zhejiang Chinese Medicine University, Hangzhou, China). The voucher specimens were deposited in the Herbarium of Zhejiang University.

These drugs were initially soaked in water for 30 min, followed by decoction in three separate stages: the first for 1.5 h, the second for 1 h, and the final for 0.5 h. The resulting decoctions were combined, filtered, and the filtrate was concentrated to a relative density of approximately 1.10 at 60 °C. After cooling, ethanol was added to induce precipitation, targeting an alcohol concentration of 60%, and the mixture was allowed to stand for over 12 h. The supernatant was collected, the ethanol was recovered, and the solution was further concentrated to achieve a medicinal material content of 2.25 g/ml. The TTF crude extract was prepared by Zhejiang Huisong Pharmaceutical Co., Ltd (ISO9001-certified).

### TTF dose confirmation

In our prior published study, AS mice were administered TTF at two doses: a low dose (0.6 g/kg/d) and high dose (2.25 g/kg/d) via gavage. The results indicated that the high-dose TTF (2.25 g/kg/d) exerted the most potent anti-atherosclerotic effects (7). In our most recently published study, we further investigated the anti-atherosclerotic effects of medium-dose (1.125 g/kg/d) and high-dose (2.25 g/kg/d) TTF. The results similarly revealed that the high-dose TTF (2.25 g/kg/d) exhibited the most potent efficacy against atherosclerosis (8).

Based on these findings, we selected 2.25 g/kg/day as the optimal dose for subsequent murine experiments. For rat studies, the equivalent dose was calculated using body surface area (BSA) normalization, yielding an adjusted dosage of 1.56 g/kg/d.

### Preparation of TTF-containing serum

Ten Sprague-Dawley rats (SPF grade, 180–220 g body weight) were acclimatized for one week prior to the experiment. The animals were then administered a crude TTF extract via gavage at a dose of 1.56 g/kg/d per administration for seven consecutive days.

One hour after the final administration, the rats were anesthetized by intraperitoneal injection of Zoletil 50 (40 mg/kg). Peripheral blood was collected via cardiac puncture and centrifuged at 3,500 rpm for 10 min to isolate serum. The serum was subsequently heat-inactivated at 56 °C for 30 min in a water bath, sterilized by filtration through a 0.22 µm membrane, and stored at −80 °C until further use.

### Identification of metabolites in TTF-containing serum

The identification of metabolites was performed on TTF crude extract, blank serum, and TTF-containing serum by UPLC-MS. The instrument was an ultra-high-performance liquid chromatography tandem high-resolution mass spectrometer.

Chromatographic column: ACQUITY UPLC HSS T3 (100 mm × 2.1 mm, 1.8 µm).

Column temperature: 45 °C.

Mobile phase: A-water (containing 0.1% formic acid) and B-acetonitrile.

Flow rate: 0.35 ml/min.

Injection volume: 5 μl.

PDA scanning range: 210–400 nm.

To ensure the accuracy of identification, the analysis was performed based on three reference criteria: (1) The retention time deviation between the sample and the reference standard in the database should be within ±0.2 min. (2) The mass error of the precursor ion (MS1) should be <5 ppm. (3) The MS/MS spectrum of the sample must match that of the reference standard.

The raw data were processed by Progenesis QI v3.0 software (Nonlinear Dynamics, Newcastle, UK) for baseline filtering, peak identification, integration, retention time correction, peak alignment and normalization. The UPLC-MS operation and data analysis were entrusted to Oebiotech Biotechnology Co., Ltd.

Automatic judgment and manual review were combined to identify blood-borne metabolites.

Automatic judgment criteria: (1) Both the TTF crude extract and the TTF-containing serum must have peaks. (2) The peak area ratio of the TTF-containing serum to the blank serum must be greater than or equal to the fold change (FC) value.

Manual review criteria: (1) TTF-containing serum and the TTF crude extract must be detected simultaneously, and the retention time must be consistent. (2) The peak area ratio of the TTF-containing serum to the blank serum was greater than or equal to the FC value, or the TTF-containing serum was detected but the blank serum was not detected.

### TTF crude extract detection procedure

The TTF crude extract (600 µl) was centrifuged at 12,000 rpm for 10 min at 4 °C. Following an overnight standing period, the sample underwent a second centrifugation under the same conditions. Subsequently, a 200 µl aliquot of the supernatant was transferred to an LC-MS vial for analysis.

### TTF-containing serum detection procedure

The TTF-containing serum (150 µl) was mixed with a protein precipitating agent consisting of methanol and acetonitrile (V:V = 2:1, 400 µl), vortexed for 1 min, and then placed in an ice water bath for 10 min to facilitate ultrasonic extraction. The sample was cooled to −40 °C for 30 min and centrifuged at 12,000 rpm at 4 °C for 10 min to separate the supernatant. The supernatant was reconstituted with a mixture of water, methanol, and acetonitrile (V: V: V = 1:2:1, 150 µl), vortexed for 1 min, sonicated for 3 min, and allowed to stand at −40 °C overnight. Following this, it was centrifuged again at 12,000 rpm at 4 °C for 10 min. Finally, a 100 µl portion of the supernatant was transferred to an LC-MS injection vial equipped with a leg-lined tube for detection.

### Experimental mice

The ApoE^−/−^ C57BL/6J mice, male, aged 6–8 weeks, weighed 21 g–27 g were procured from Jiangsu Jicui Biotechnology Co., Ltd. The animal production license number is SCXK (Su) 2023-0009. The animal experiments received approval from the Animal Ethics Committee of Zhejiang Chinese Medical University (IAUC-20241209-27). The mice were acclimated to laboratory conditions for one week prior to the commencement of the experiment and were subsequently stratified into three cohorts. The cohorts included the Control group (regular diet with saline gavage), the AS group (high-fat diet with saline gavage), and the TTF group (high-fat diet with 2.25 g/kg/d TTF crude extract gavage).

The high-fat diet consisted of 40% kcal from fat, 1.25% cholesterol, and 0.5% cholic acid (RESEARCH DIETS, New Brunswick; Cat# D12109C). The TTF crude extract was prepared by Zhejiang Huisong Pharmaceutical Co., Ltd, with a concentration of 2.25 g/ml.

At weeks 6, 10, and 12, the mice were anesthetized via intraperitoneal injection of Zoletil 50 at a dosage of 80 mg/kg, followed by orbital blood collection, and the heart, aorta, and liver were harvested for further analysis.

### Oil red O staining of the entire aorta

The entire aorta was fixed using a 4% paraformaldehyde solution (Biosharp; Cat# BL539A). Following a fixation period of 1 h, the aorta underwent staining with a modified Oil Red O staining kit (Beyotime; Cat# C0158M). After the removal of external fat from the aortic tissue, it was mounted in neutral balsam for preservation and clarity. The stained sections were subsequently examined using a high-resolution digital pathology slide scanner (Panoramic MIDI, 3DHISTECH Ltd). The area of atherosclerotic plaques was quantified using Image J software.

### Aortic valve sectioning

The heart was fixed in 4% paraformaldehyde (Biosharp; Cat# BL539A) for 1 h. Following fixation, the tissue was dehydrated in a graded sucrose series, first in 20% sucrose and then in 30% sucrose, until it reached sufficient density to sink. The heart was then embedded in Optimal Cutting Temperature (OCT) compound. Serial sections of the aortic valve were cut at a precise thickness of 8 μm, ensuring high-resolution preservation for downstream analysis.

### Oil red O staining of the aortic valve

The initial three consecutive sections adjacent to the aortic valve were selected for Oil Red O staining. These sections were immersed in the Oil Red O solution for 30 min to facilitate the staining process. Subsequently, the cell nuclei were accentuated with hematoxylin counterstaining for 1 min, enhancing the contrast between the lipid-rich areas and the nuclei. Upon completion of the staining process, the sections were mounted using a glycerin gelatin medium. The mounted sections were then digitally scanned with a high-resolution pathology slide scanner (Panoramic MIDI, 3DHISTECH Ltd). Image J software was utilized to measure both the area of the atherosclerotic plaques and the total area of the aortic valve sections.

### Masson staining of the aortic valve

The fifth section adjacent to the aortic valve was specifically selected for Masson staining (Pinophy; Cat# S191006). The sections were incubated overnight in Masson stain solution 1, rinsed with water until colorless, and preheated at 65 °C for 30 min. They were then stained in Masson stain solution 2 for 3–5 min, followed by 2–3 water rinses. Next, the sections were immersed in Masson stain solution 3 for 1 min, drained, and preheated again at 65 °C for 30 min before being stained in Masson stain solution 4 for 5–20 s. The sections were then placed in 1% glacial acetic acid (three consecutive baths, 10 s each), dehydrated in absolute ethanol, transferred to n-butanol for 10–20 s, cleared in xylene I (5 min) and xylene II (5 min), air-dried rapidly, and finally mounted with neutral balsam. Image J software was employed to quantify the collagen area within the plaques.

### Prediction of potential targets for TTF in as treatment

Potential targets of TTF blood-borne metabolites were predicted using the SwissTargetPrediction database (http://www.swisstargetprediction.ch) with stringent selection criteria (probability score ≥0 and top 100 ranking). After removing duplicates, 591 unique targets were identified, excluding Yuehgesin C which showed no target associations. Furthermore, the TTD database (https://db.idrblab.net/ttd/), DrugBank database (https://go.drugbank.com), KEGG database (https://www.kegg.jp), and OMIM database (https://omim.org) were leveraged to predict AS-related targets and yielded 504 distinct AS-related targets. The intersection between TTF-related targets and AS-related targets was analyzed to identify overlapping genes. The STRING database (https://www.string-db.org) was employed to identify pivotal targets and their relationships. The protein-protein interaction (PPI) network was visualized and analyzed using Cytoscape software (Version 3.8.2), providing insights into the complex target interplay relevant to TTF's therapeutic effects on AS. Key genes were subsequently identified through the Maximal Clique Centrality (MCC) algorithm.

### Gene functional enrichment analysis

GO enrichment analysis was conducted to elucidate the functions of genes and proteins by linking them to specific GO terms. Furthermore, KEGG enrichment analysis was utilized to categorize and enrich genes and proteins based on pathway annotation data. These enrichment analyses were performed using R software (Version 4.2.1) in conjunction with the clusterProfiler package (Version 4.4.4).

### Liver HE staining

The liver was fixed in 4% paraformaldehyde (Biosharp; Cat# BL539A) for 24 h, after which it was transferred to a 15% sucrose solution for dehydration for 1 day. Following this, the liver was moved to a 30% sucrose solution for additional dehydration for another day. After dehydration, the liver was embedded in OCT compound, and sections were cut to a thickness of 8 µm. These sections were then fixed in methanol for 15 min before undergoing HE staining, and subsequently sealed with neutral resin.

### Liver transcriptome sequencing

To investigate TTF's mechanisms, livers from the Control, AS, and TTF groups (*n* = 4 each) underwent transcriptome sequencing for pathway analysis. The screening criteria: *P* < 0.05, |log_2_FC| > 1. Transcriptome sequencing was performed by Gene Denovo Biotechnology Co., Ltd. Venn diagrams were used to identify overlapping genes. GO and KEGG analyses were conducted for gene function enrichment.

### Lipid metabolomics analysis

Targeted lipidomic profiling of serum was performed using UPLC-MS/MS technology. The Thermo Ultimate 3000 ultra-high-performance liquid chromatography coupled with TSQ Endura MD Plus triple quadrupole mass spectrometry was utilized to detect 722 types of lipids. Targeted lipidomic profiling was conducted by Hangzhou Hanku Medical Laboratory.

### Isolation of peritoneal macrophages (PMs) from mice

C57BL/6J mice were intraperitoneally injected with 1 ml of 3% thioglycollate broth once daily for three consecutive days. After anesthesia with Zoletil 50, the mice were euthanized by cervical dislocation and sterilized by immersion in 75% ethanol for 3–5 min. The mice were then fixed in a supine position, and the peritoneum was exposed. A total of 5 ml of ice-cold RPMI-1640 medium supplemented with 10% serum was slowly injected into the lower right abdominal cavity. The abdomen was gently massaged several times, and the fluid was allowed to circulate within the peritoneal cavity for 3–5 min. The peritoneal lavage fluid was aspirated and transferred to a centrifuge tube (the peritoneal cavity was rinsed twice to maximize cell recovery).

The isolated PMs were identified by immunofluorescence staining. Cells were incubated overnight at 4 °C with a primary antibody (F4/80; BioLegend), followed by PBS washes and incubation with a fluorophore-conjugated secondary antibody for 1 h in the dark. Nuclei were counterstained with DAPI (10 min). Images were captured using a confocal laser scanning microscope (Zeiss LSM 880). Macrophage purity was considered high when over 90% of cells were F4/80-positive (Supplementary Figure 1).

### *In vitro* cell experiments

PMs were pretreated with 10% TTF-containing serum for 1 h, followed by stimulation with LPS (0–10 μg/ml) for various durations (2, 4, 6, 12, and 24 h). Cell viability was assessed using CCK-8 assays (Beyotime; Cat# C0038) to determine the optimal LPS concentration and treatment duration.

Under these optimized conditions, PMs were pretreated with different concentrations of TTF-containing serum (5%, 10%, 15%, and 20%), and cell viability was again evaluated by CCK-8 assay to identify the optimal drug concentration.

For subsequent experiments, PMs were pre-treated with 5% or 10% TTF-containing serum for 1 h, followed by stimulation with 2 μg/ml LPS for 6 h. After treatment, cells were washed twice with ice-cold PBS and harvested for RT-PCR and WB analyses.

### RT-PCR

Total RNA was extracted from PMs using Trizol reagent (Invitrogen; Cat# 65307-12-2) followed by chloroform-phenol extraction. RNA purity was assessed by measuring the A260/A280 ratio (1.8–2.0) using a spectrophotometer (NanoDrop, Thermo Fisher Scientific). Reverse transcription was performed using the Hifair III 1st Strand cDNA Synthesis SuperMix for qPCR (gDNA digester plus, YEASEN; Cat# 11141ES60).

PCR amplification was carried out with Hieff UNIcoN® Universal Blue qPCR SYBR Green Master Mix (YEASEN; Cat# 11184ES08) under the following conditions:

1. Initial denaturation: 95 °C for 2 min (1 cycle)
2. Amplification: 95 °C for 10 s, 60 °C for 30 s (40 cycles)
3. Melting curve analysis

IL-6 Primer Sequences:

Forward (5′-3′): TAGTCCTTCCTACCCCAATTTCC

Reverse (5′-3′): TTGGTCCTTAGCCACTCCTTC.

IL-10 Primer Sequences:

Forward (5′-3′): GGTTGCCAAGCCTTATCGGA

Reverse (5′-3′): AGACACCTTGGTCTTGGAGCTTA.

β-actin Primer Sequences:

Forward (5′-3′): GAGATTACTGCCCTGGCTCCTAGC

Reverse (5′-3′): CCGGACTCATCGTACTCCTGCTT.

### WB

Total protein was extracted using RIPA lysis buffer (Fdbio science; Cat# FD008) and quantified using the BCA assay kit (Beyotime; Cat# P0012S). After adjusting the concentration with protein loading buffer (Beyotime; Cat# P0015), the samples were fully denatured at 95 °C for 10 min. The denatured samples were then separated by 10% SDS-PAGE and transferred onto a PVDF membrane. The membrane was blocked with 5% skim milk (Biosharp; Cat# BS102) for 1 h and incubated with the primary antibody against the target protein at 4 °C for 18 h. After washing, it was incubated with the secondary antibody at room temperature for 90 min. Following further washing, the membrane was developed with ECL reagent (Biosharp; Cat# BL520A) and the protein signals were detected using a gel imaging system (Bio-rad, USA).

The primary antibodies included NLRP3 Recombinant Rabbit mAb (1:1,000, Diagbio; Cat# db12063), IL-18 Recombinant Rabbit mAb (1:1,000, Diagbio; Cat# db15266), IL-1β Recombinant Rabbit mAb (1:1,000, Diagbio; Cat# dbdb12096), IL-6 Recombinant Rabbit mAb (1:1,000, Diagbio; Cat# db9181). The secondary antibody was Goat Anti Rabbit IgG (H + L)-HRP (1:5,000, Diagbio; Cat# db10002).

### Detection of Il-6 and Il-1β

Peripheral blood was collected and centrifuged at 3,500 rpm for 10 min to isolate serum. The serum was used to measure IL-6 and IL-1β using commercially available ELISA kits as follows:

IL-6: Mouse IL-6 ELISA Kit (Jianglai Biotechnology; Cat# JL20268).

IL-1β: Mouse IL-1β ELISA Kit (Jianglai Biotechnology; Cat# JL18442).

The enzyme-linked immunosorbent assay was the Cmax Plus model (Molecular Devices, Hangzhou, China).

### Statistical analysis

For comparisons between two groups, a *T*-test was selected if the data were normally distributed and exhibited homogeneity of variance. The Welch's *t*-test was utilized if the data were normally distributed but did not demonstrate homogeneity of variance. The Wilcoxon rank sum test was used if the data were not normally distributed.

For comparisons among multiple groups, a One-way ANOVA was chosen if the data were normally distributed and exhibited homogeneity of variance. The Welch's one-way ANOVA was applied if the data were normally distributed but did not exhibit homogeneity of variance. The Kruskal–Wallis test was choosed if the data were not normally distributed.

The statistical analysis and data visualization were conducted using R software (Version 4.2.1), along with the ggplot2 package (Version 3.3.6), the stats package (Version 4.2.1), and the car package (Version 3.1-0).

## Results

### TTF identification and quality control

**Figure 1:**
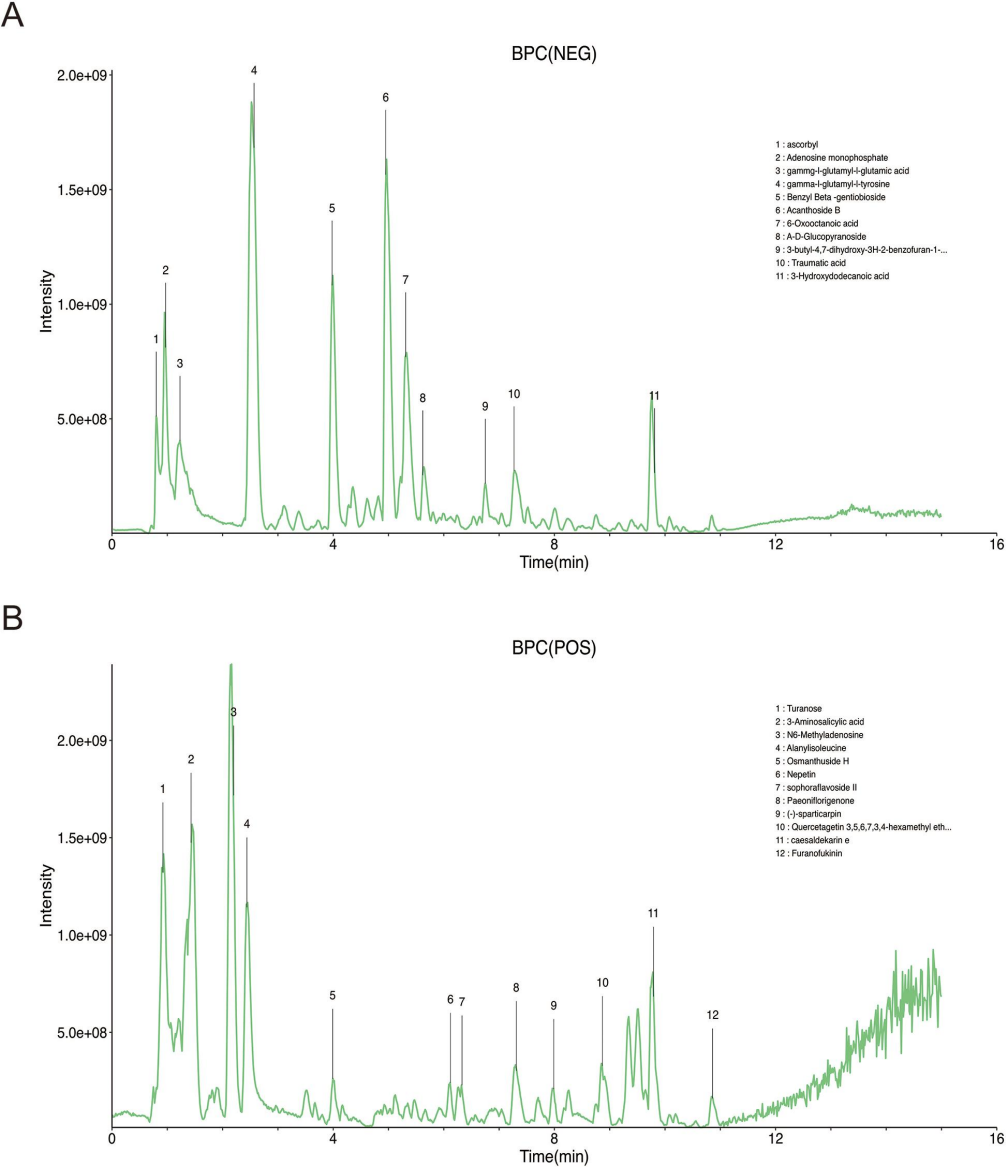
Identification of TTF crude extract by UPLC-MS **(A)** chromatogram of TTF in the negative mode of BPC. **(B)** Chromatogram of TTF in the positive mode of BPC. TTF, Tanyu Tongzhi Formula, UPLC-MS, ultra performance liquid chromatography-mass spectrometry; BPC, base peak chromatogram.

Based on accurate mass, secondary fragmentation, isotope distribution and TCM database, the compounds in the TTF crude extract were identified by UPLS-MS/MS. The Base Peak Chromatogram (BPC) of the TTF crude extract was shown in Figures 1A,B. The quality of TTF crude extract met the requirements of the Pharmacopoeia of the People's Republic of China in 2020.

### Identification of TTF-containing serum and functional enrichment analysis

**Figure 2:**
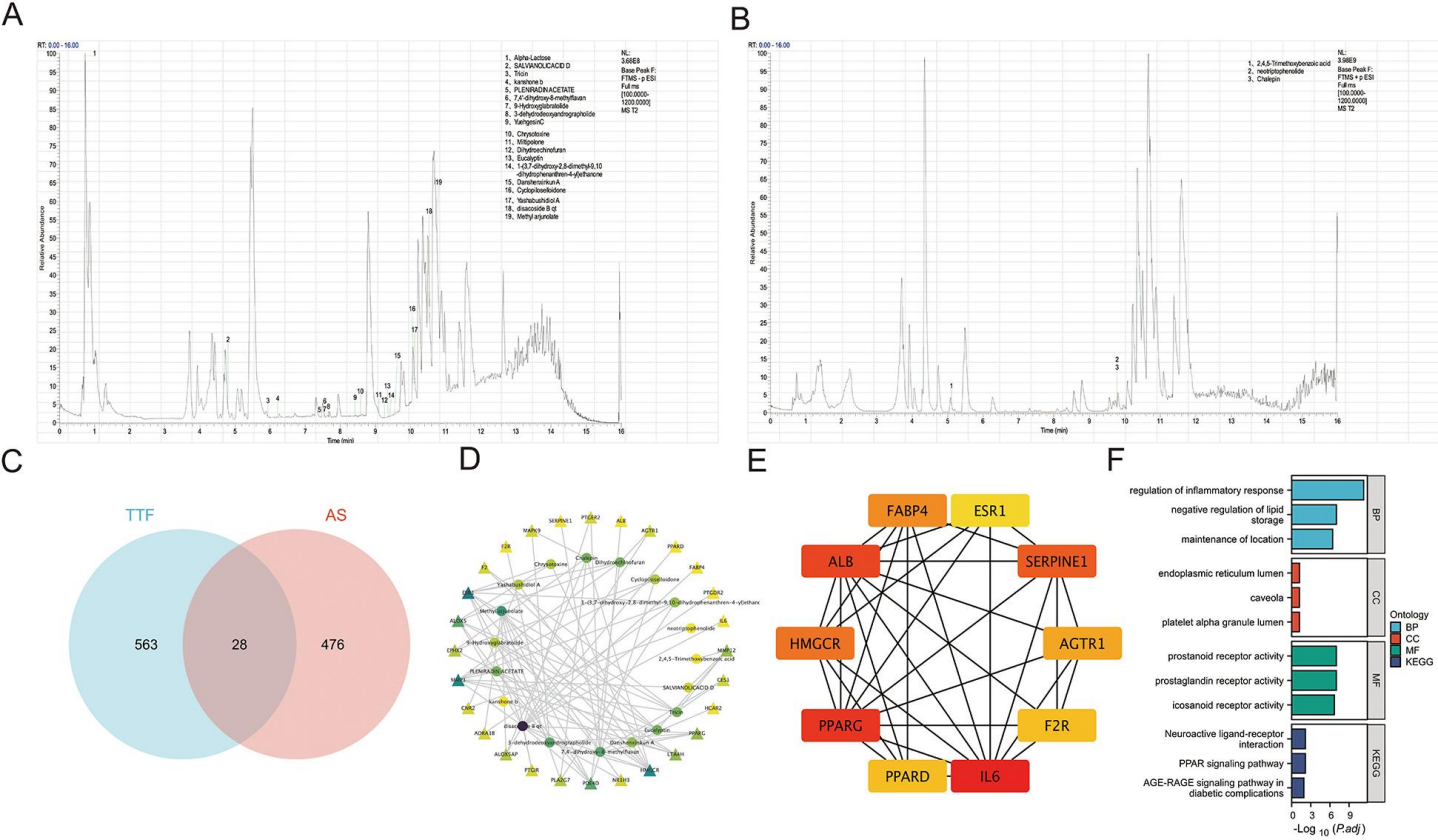
Identification of TTF-containing serum and network analysis. **(A)** Chromatogram of TTF-containing serum in the negative mode. **(B)** Chromatogram of TTF-containing serum in the positive mode. **(C)** Venn diagram of TTF-related targets and AS-related targets. **(D)** The network of the relationship between blood-borne metabolites and AS-related targets. **(E)** Top ten genes identified by the MCC algorithm. **(F)** GO and KEGG pathway enrichment analysis on these overlapping genes. Prostanoid receptor activity (GO: 0004954): Combining with a prostanoid, any compound based on or derived from the prostanoate structure, to initiate a change in cell activity. Prostaglandin receptor activity (GO: 0004955): Combining with a prostaglandin to initiate a change in cell activity. Icosanoid receptor activity (GO: 0004953): combining with an icosanoid to initiate a change in cell activity. TTF, Tanyu Tongzhi Formula; AS, atherosclerosis; MCC, multiple comparison correction; GO, gene ontology; KEGG, Kyoto encyclopedia of genes and genomes; BP: biological process; CC, cellular component; MF, molecular function.

**Figure 3:**
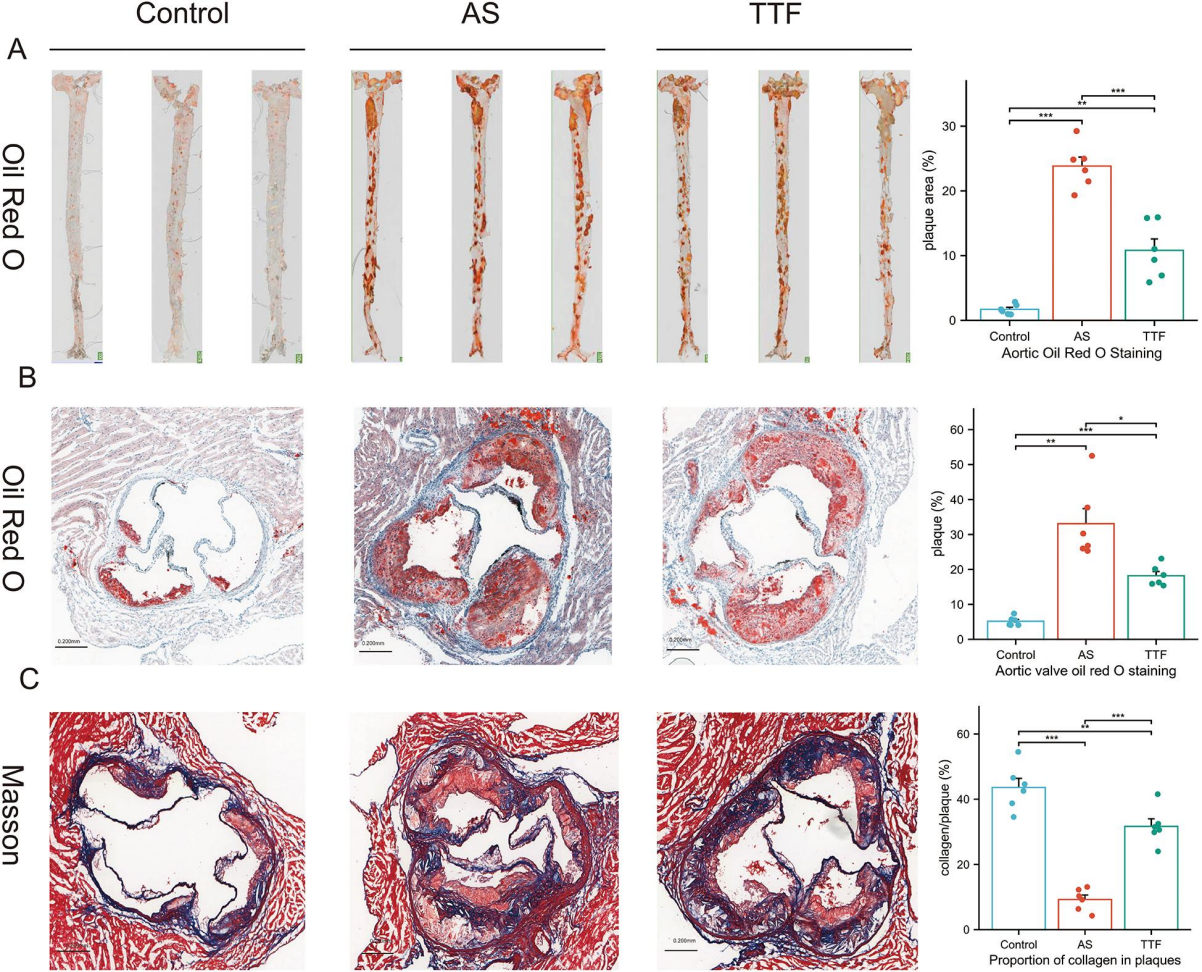
TTF suppresses atherosclerotic plaque progression. **(A)** Oil red O staining of the aorta. **(B)** Oil red O staining of the aortic valve. **(C)** Masson staining of the aortic valve. TTF: Tanyu Tongzhi Formula; AS, atherosclerosis. *: *P* < 0.05, **: *P* < 0.01, ***, *P* < 0.001.

A total of 22 metabolites were identified in TTF-containing serum using UPLC-MS/MS, comprising 19 metabolites detected in negative ionization (NEG) mode (Figure 2A) and 3 metabolites detected in positive ionization (POS) mode (Figure 2B). To predict AS-related targets, we queried the TTD, DrugBank, KEGG, and OMIM databases, yielding 504 potential AS-associated targets. Additionally, the SwissTargetPrediction database was utilized to identify molecular targets for the 22 blood-borne metabolites (Table 2), yielded 591 predicted targets. A Venn diagram analysis was performed to identify overlapping targets between the disease and metabolite-related targets, revealing 28 overlapping genes (Figure 2C). The interaction network of TTF-AS targets was visualized using Cytoscape software (Figure 2D). To prioritize these targets, we applied the MCC algorithm, which ranked the top ten targets based on their scores in descending order: IL-6, PPARG, ALB, SERPINE1, HMGCR, FABP4, AGTR1, F2R, PPARD, and ESR1 (Figure 2E).

**Table 2:** Identification of the metabolites in TTF-containing serum.

No.	Formula	Metabolites	Theoretical (m/z)	Retention time (min)	Ion mode	FoldChange
1	C10H12O5	2,4,5-Trimethoxybenzoic acid	213.0758	5.11	POS	333.25
2	C12H22O11	Alpha-Lactose	387.1144	0.74	NEG	32,768.00
3	C18H16O4	Danshenxinkun A	295.0976	9.54	NEG	32,768.00
4	C15H22O4	kanshone b	265.1445	6.21	NEG	32,768.00
5	C31H50O5	Methyl arjunolate	483.348	10.74	NEG	32,768.00
6	C19H24O3	Miltipolone	345.1708	9.09	NEG	32,768.00
7	C21H26O4	neotriptophenolide	360.217	9.78	POS	500.54
8	C17H22O5	PLENIRADIN ACETATE	305.1394	7.4	NEG	32,768.00
9	C20H18O10	SALVIANOLICACID D	417.0827	4.8	NEG	32,768.00
10	C17H14O7	Tricin	311.0561	5.95	NEG	32,768.00
11	C19H24O2	Yashabushidiol A	329.1758	10.12	NEG	32,768.00
12	C17H22O5	YuehgesinC	287.1289	8.42	NEG	32,768.00
13	C19H18O5	Eucalyptin	325.1081	9.36	NEG	114.51
14	C18H18O3	1-(3,7-dihydroxy-2,8-dimethyl-9,10-dihydrophenanthren-4-yl) ethanone	281.1183	9.4	NEG	32,768.00
15	C20H28O4	3-dehydrodeoxyandrographolide	331.1915	7.62	NEG	32,768.00
16	C16H16O3	7,4′-dihydroxy-8-methylflavan	301.1082	7.56	NEG	32,768.00
17	C19H24O6	9-Hydroxyglabratolide	329.1394	7.56	NEG	94.11
18	C18H20O5	Dihydroechinofuran	297.1132	9.27	NEG	32,768.00
19	C30H48O4	disacoside B qt	471.348	10.54	NEG	32,768.00
20	C18H22O3	Cyclopiloselloidone	331.1551	10.06	NEG	32,768.00
21	C18H22O5	Chrysotoxine	317.1394	8.57	NEG	32,768.00
22	C19H22O4	Chalepin	337.1411	9.78	POS	2,154.33

GO and KEGG pathway enrichment analyses of the 28 overlapping genes demonstrated that the BP were primarily enriched in “regulation of inflammatory response,” “negative regulation of lipid storage,” among others (Figure 2F).

### Validation of TTF's therapeutic effects in animal models

Aortas and aortic valves were carefully dissected from Control, AS, and TTF groups (*n* = 6 per group) for quantitative analysis of atherosclerotic plaques and collagen deposition. Oil Red O staining revealed significantly larger plaque areas in both AS and TTF groups compared with Controls (*P* < 0.05). Importantly, TTF treatment significantly reduced plaque area compared with AS group (*P* < 0.05) (Figures 3A,B). Masson staining showed markedly decreased collagen content in AS group plaques vs. Controls (*P* < 0.05). TTF administration significantly increased collagen content compared with AS group (*P* < 0.05) (Figure 3C).

### Liver HE staining and transcriptome sequencing

**Figure 4:**
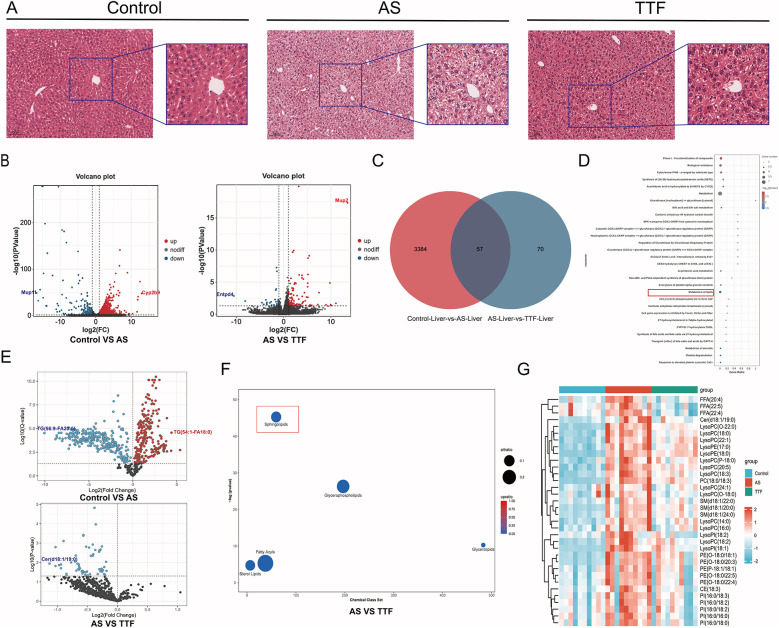
Regulation of lipid metabolism by TTF. **(A)** HE staining of the livers. **(B)** Volcano plot of DEGs in liver transcriptome. **(C)** Venn diagram of DEGs between AS and TTF groups. **(D)** GO and KEGG pathway enrichment analysis on these overlapping genes. **(E)** Volcano plot of differential lipids in plasma lipidomic profile. **(F)** Chemical enrichment analysis on differential lipid metabolites between AS group and TTF group. **(G)** The heatmap of differential lipids among control, AS, and TTF groups. TTF: Tanyu Tongzhi Formula; DEGs, differentially expressed genes; AS, atherosclerosis; GO, gene ontology; KEGG, Kyoto encyclopedia of genes and genomes. DEGs screening threshold: *P* < 0.05, |log_2_FC| > 1. Differential lipid threshold: Q < 0.05. ns: *P* > 0.05, **: *P* < 0.01, ***, *P* < 0.001.

HE staining revealed that livers in the AS group exhibited vacuolation, with the vacuoles in the TTF group appearing lighter than those in the AS group (Figure 4A). Transcriptome sequencing of the livers identified 3,441 differentially expressed genes (DEGs) between the Control group and the AS group, as well as 127 DEGs between the AS group and the TTF group (Figure 4B). Venn analysis of these DEGs yielded 57 overlapping genes (Figure 4C). Gene Set Enrichment Analysis on these 57 overlapping genes mainly focused on “Metabolism”, and “Metabolism of lipids”, among others (Figure 4D).

### Lipid metabolomics

Serum from the Control group, AS group, and TTF group was collected for targeted lipidomics analysis. Based on *Q* < 0.05, the primary differences of the lipids were identified between the AS and Control groups, as well as between the TTF and AS groups (Figure 4E). Notably, compared with the AS group, all lipids that differed in the TTF group exhibited down-regulation. The ChemRICH algorithm was utilized for chemical enrichment analysis. The differential lipids between the TTF and AS groups were primarily concentrated in Sphingolipids, Glycerophospholipids, Fatty Acyls, Sterol Lipids, and Glycerolipids (Figure 4F). Among them, sphingolipids showed the most significant difference. A total of 34 differential lipids elevated in the AS group, but decreased simultaneously in the TTF group (*P* < 0.05) (Figure 4G).

### Detection of Il-6 and Il-1β in peripheral blood

**Figure 5:**
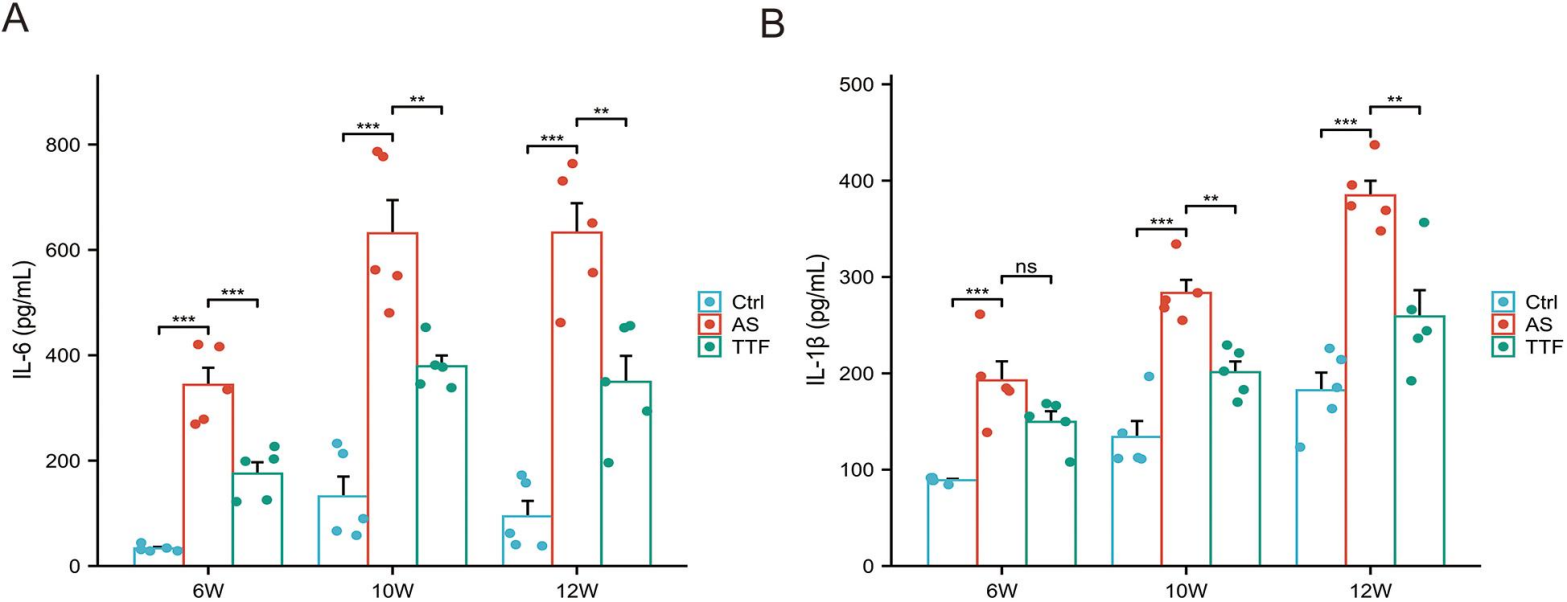
IL-6 and IL-1β levels in peripheral blood. **(A)** IL-6 levels in peripheral blood post-TTF treatment (weeks 6/10/12). **(B)** IL-1β levels in peripheral blood post-TTF treatment (weeks 6/10/12). ns: *P* > 0.05, **: *P* < 0.01, ***, *P* < 0.001.

Peripheral blood was collected from ApoE^−/−^ C57BL/6J mice at weeks 6, 10, and 12 for quantification of IL-6 and IL-1β. Compared with the Control group, those fed on a high-fat diet exhibited significantly elevated circulating levels of both IL-6 and IL-1β (*P* < 0.05). High-fat diet significantly increased circulating IL-6/IL-1β vs. regular diet. TTF consistently reduced IL-6 (weeks 6, 10, 12) and significantly lowered IL-1β at later timepoints (weeks 10, 12) (*P* < 0.05), with only marginal week-6 effects (*P* > 0.05) (Figures 5A,B).

### TTF inhibits inflammation *in vitro*

**Figure 6:**
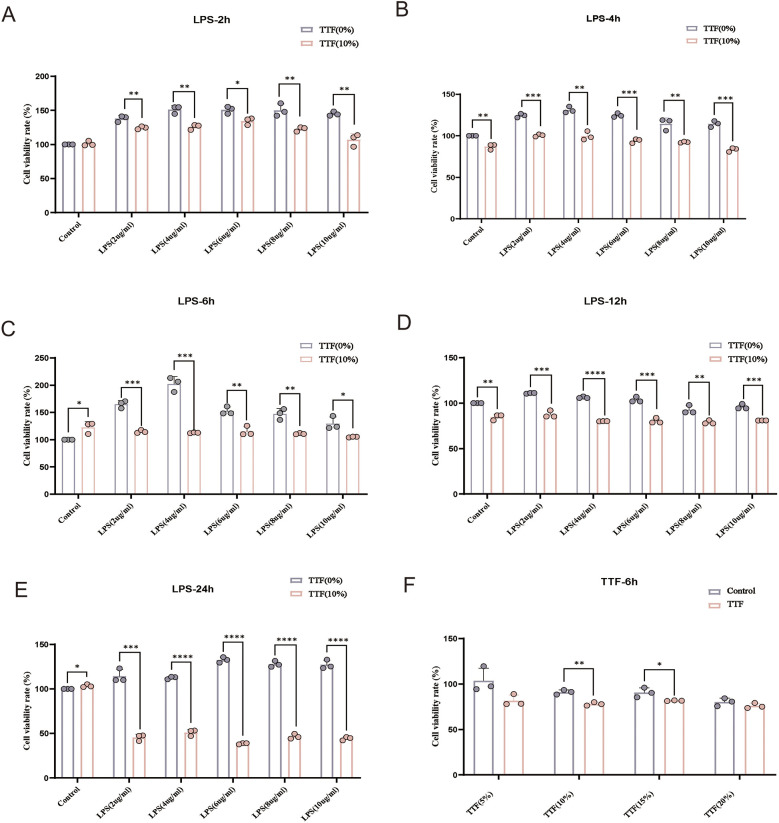
Detection of viability in LPS and TTF-treated PMs by CCK-8. **(A–E)** Cell viability of PMs treated with different concentrations of LPS for 2, 4, 6, 12, and 24 h. **(F)** Cell viability of PMs pretreated with different concentrations of TTF-containing serum, followed by 2 μg/ml LPS stimulation for 2 h. PMs: peritoneal macrophages. TTF, Tanyu Tongzhi Formula. *: *P* < 0.05, **: *P* < 0.01, ***, *P* < 0.001.

PMs were stimulated with 0–10 μg/ml LPS and treated with 10% TTF-containing serum for 2, 4, 6, 12, and 24 h. Compared with the Control group, 2 μg/ml LPS significantly increased cell viability. TTF counterbalanced the LPS-induced increase in cell viability, and after 6 h, the cell viability of TTF-treated PMs returned to near-normal levels (Figures 6A–E). Based on these findings, the optimal LPS concentration was determined to be 2 μg/ml, with an intervention time of 6 h. Under these conditions, PMs were treated with 5%–20% TTF-containing serum, and the results showed that 10% TTF significantly reduced LPS-induced cell viability (Figure 6F).

**Figure 7:**
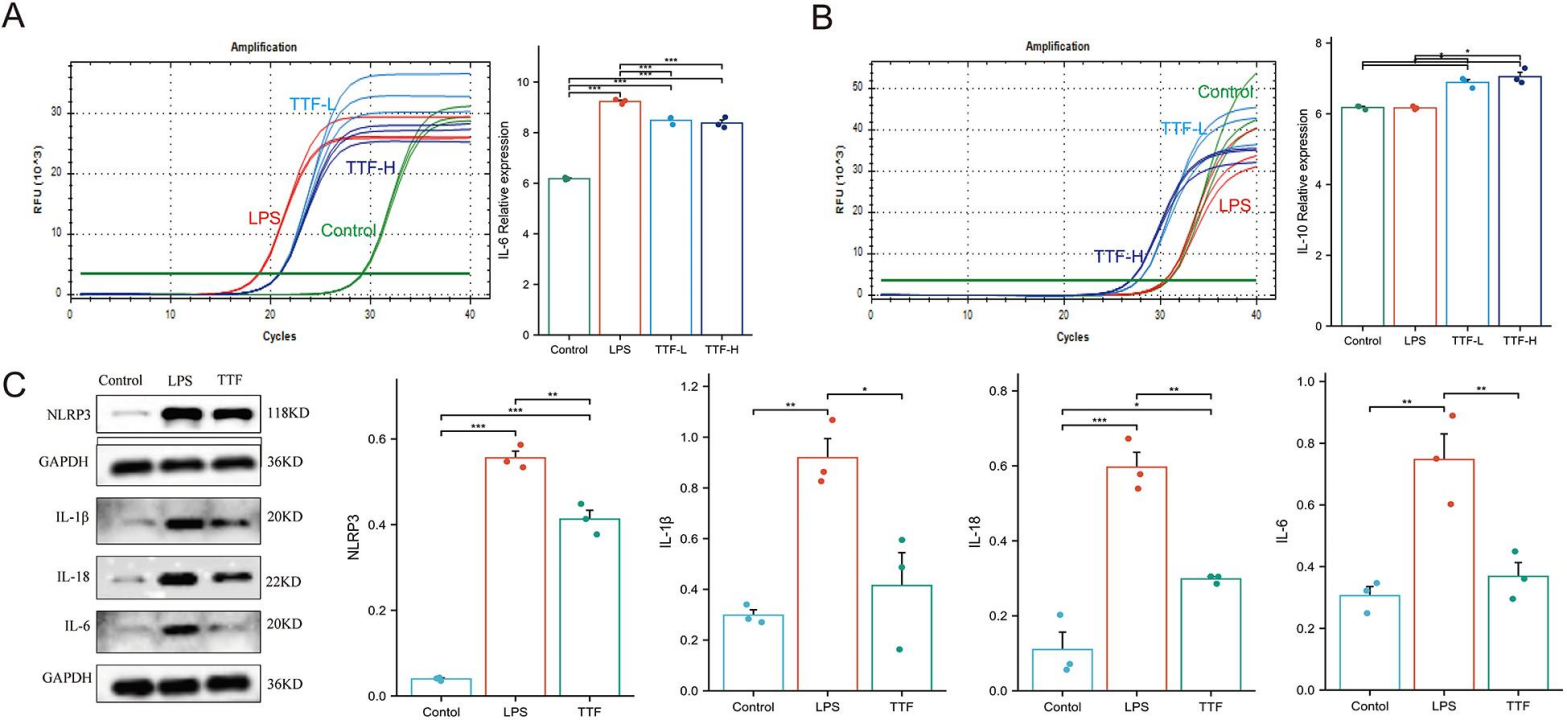
Cellular experiments validating TTF-mediated inflammation regulation. **(A)** TTF modulates IL-6 mRNA expression in LPS-stimulated PMs. **(B)** TTF modulates IL-10 mRNA expression in LPS-stimulated PMs. **(C)** TTF alters inflammasome-related protein expression in LPS-stimulated PMs. PMs: peritoneal macrophages. TTF, Tanyu Tongzhi Formula. TTF-L: 5% TTF-containing serum. TTF-H & TTF: 10% TTF-containing serum. *: *P* < 0.05, **: *P* < 0.01, ***: *P* < 0.001.

PMs were pretreated with either low (5%) or high (10%) concentration of TTF-containing serum for 1 h, followed by stimulation with 2 μg/ml LPS for 6 h. RT-PCR analysis revealed that LPS stimulation significantly upregulated IL-6 mRNA expression in PMs (*P* < 0.05). Notably, compared with the LPS-treated group, both 5% and 10% TTF-containing serum significantly reduced IL-6 expression while simultaneously enhancing IL-10 mRNA levels (Figures 7A,B). Both 5% and 10% TTF-containing serum showed equivalent efficacy in suppressing IL-6, while 10% TTF serum slightly enhanced IL-10 expression (*P* > 0.05).

To further investigate the anti-inflammatory mechanism of TTF, PMs were pretreated with 10% TTF-containing serum for 1 h prior to LPS stimulation (2 μg/ml, 6 h). WB analysis demonstrated that LPS significantly elevated the protein levels of key inflammasome components including NLRP3, IL-1β, IL-18, and IL-6 compared to the control group (*P* < 0.05). Importantly, TTF pretreatment effectively suppressed the LPS-induced upregulation of these inflammatory mediators (*P* < 0.05, Figure 7C).

## Discussion

AS is a lipid-driven inflammatory arterial disease characterized by the formation and gradual enlargement of plaques within the arterial intima (9). These plaques can lead to arterial narrowing, increased resistance to blood flow, and potentially trigger thrombosis, thereby contributing to ASCVD (10). The instability of these plaques serves as a critical marker for the progression of AS and is closely associated with the risk of ASCVD (11). TCM plays a significant clinical role in the prevention and treatment of AS (12, 13). Its multi-components, multi-targets, and multi-pathways can effectively inhibit the progression of AS, reduce the likelihood of plaque rupture, and consequently decrease the incidence of ASCVD (14).

TTF, as an empirical formula derived from the clinical experience of renowned TCM experts, has been clinically proven to improve cardiac function in patients with CHD (15). In our preliminary study, ApoE^−/−^ mice fed a high-fat diet were used to establish the AS model, and atorvastatin (10 mg/kg/day) served as the positive control. Notably, high-dose TTF (2.25 g/kg/d) reduced both plaque and necrotic core areas to levels comparable with those achieved by atorvastatin (8). However, the active metabolites of TTF entering the blood were unclear, and the therapeutic mechanisms warranted further investigation. The application of modern technology aided in elucidating the effective metabolites and mechanisms of TTF in treating AS, thereby facilitating its clinical application.

In this study, we successfully prepared TTF-containing serum and identified 22 metabolites in TTF-containing serum using UPLC-MS technology. Through systematic target prediction analysis of these 22 metabolites, we identified 28 potential molecular targets of TTF for AS treatment. Functional enrichment analysis demonstrated significant clustering of these targets in inflammation and lipid metabolism. Inflammatory plays a crucial role in the initiation, development, and complications of AS (16). Inflammatory mediators can attract immune cells to the vascular wall, resulting in the infiltration of inflammatory cells and the formation of plaques (17). By using the MCC algorithm in Cytoscape software, we obtained the top ten potential targets of TTF for the treatment of AS. Among them, IL-6 ranks first.

IL-6, as an important inflammatory factor, is closely related to the occurrence and development of AS (18). Based on these computational findings, we subsequently validated TTF's regulatory effects on both inflammatory responses and lipid metabolism through integrated animal models and cellular experiments. In animal experiments, we confirmed that TTF could reduce the levels of IL-6 and IL-1β in the serum of ApoE^−/−^ AS mice. Major trials (CANTOS, COLCOT, LoDoCo2, RESCUE) established that anti-inflammatory therapy targeting IL-1β or IL-6 significantly lowers MACE risk in CAD patients (19–22). Subsequently, we examined the regulatory effects of TTF on lipid metabolism in ApoE^−/−^ AS mice. HE staining of livers demonstrated that TTF significantly ameliorated hepatic steatosis in AS mice. RNA-seq analysis uncovered TTF-mediated modulation of lipid metabolism. Targeted lipidomic analysis indicated that TTF effectively decreased serum lipid levels in AS mice compared with the AS group, with sphingolipids being the most prominently altered lipid class. Sphingolipids are a class of bioactive lipids, including ceramides and sphingomyelins. Among them, ceramides and sphingomyelins act as key regulators of pro-inflammatory factors and serve as driving forces in AS (23). Recent studies have identified ceramides as potential therapeutic targets for CVD (24). Inhibiting sphingolipid metabolism can help restrict inflammatory responses (25). Specifically, targeting serine-palmitoyltransferase (SPT) to suppress the *de novo* synthesis pathway of ceramides may alleviate AS (26). Therefore, we hypothesized that TTF might mitigate AS by modulating sphingolipid metabolism and subsequently suppressing inflammation.

*In vitro*, we established an inflammatory model by stimulating PMs with LPS and found that TTF suppressed IL-6 mRNA expression while promoting IL-10 mRNA. Additionally, TTF inhibited inflammasome activation. IL-10 is a classic anti-inflammatory cytokine that plays a critical role in modulating immune inflammation in AS, whereas IL-6 is a key mediator of systemic inflammation (27). Elevated IL-6 levels significantly increase the risk of CAD and promote AS progression. Through *in vitro* experiments, we further confirmed that TTF markedly suppresses inflammatory responses.

In summary, our integrated approach combining bioinformatics, multi-omics analyses, animal experiments, and cellular assays demonstrates that TTF alleviates atherosclerotic plaque progression by regulating lipids and suppressing inflammation. However, our study has several limitations. While our data demonstrate TTF's dual effects on lipid modulation and inflammatory suppression in murine AS models, the causal relationship between these two mechanisms remains unvalidated. Furthermore, all animal experiments were conducted exclusively in male mice to minimize the metabolic variability associated with the estrous cycle in females, a approach consistent with common practice in preliminary pharmacological studies. While this design enhances internal validity, it precludes any conclusions regarding the efficacy of TTF in female animals. Thus, dedicated investigation into the sex-specific effects of TTF is warranted in future research. Although the anti-inflammatory efficacy of TTF was consistently observed across both cellular (LPS-stimulated macrophages) and animal (AS mice) models, the current findings lack clinical translation, as no human trial data were included to corroborate these preclinical results.

## Conclusions

In our study, UPLC-MS was used to identify the metabolites of TTF entering the bloodstream. Through network analysis, potential molecular targets and pathways of action for TTF treatment of AS were identified, suggesting that TTF may alleviate AS by inhibiting inflammation and regulating lipid metabolism. In animal experiments, we further confirmed that TTF may reduce pro-inflammatory cytokine levels in AS mice and inhibit the progression of aortic plaques. By stimulating PMs with LPS to construct an inflammatory model, we confirmed the inhibitory effect of TTF on inflammation at the cellular level. Inflammation, as a risk factor for the progression of AS. Therefore, we infer that TTF can alleviate AS by inhibiting inflammatory response.

## Data Availability

The raw data supporting the conclusions of this article will be made available by the authors, without undue reservation.

## Supplementary material

Supplementary Figure S1 Identification of PMs through immunofluorescence. PMs: peritoneal macrophages.
