# Supplementary figures and images for: Tanyu Tongzhi Formula relieves the progression of atherosclerotic plaque through lipid regulation and anti-inflammatory effects
Source: Front Cardiovasc Med. 2025 Sep 29;12:1614525. doi: 10.3389/fcvm.2025.1614525 (PMC12515906; doi:10.3389/fcvm.2025.1614525)

WB raw data

NLRP3  
118KD

GAPDH  
36KD

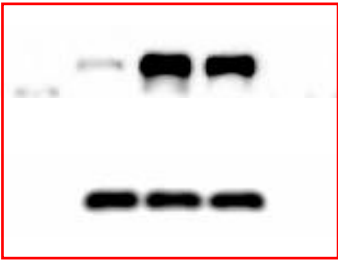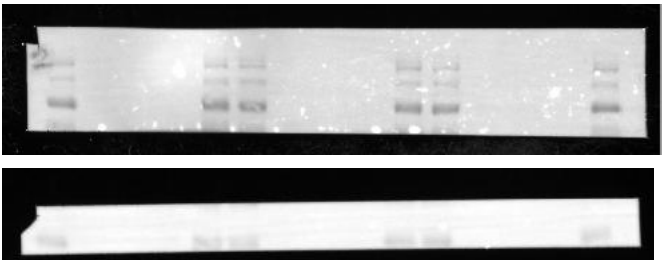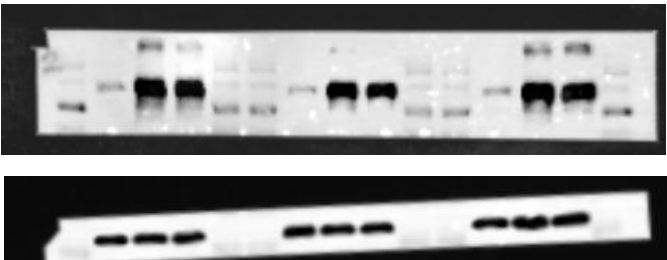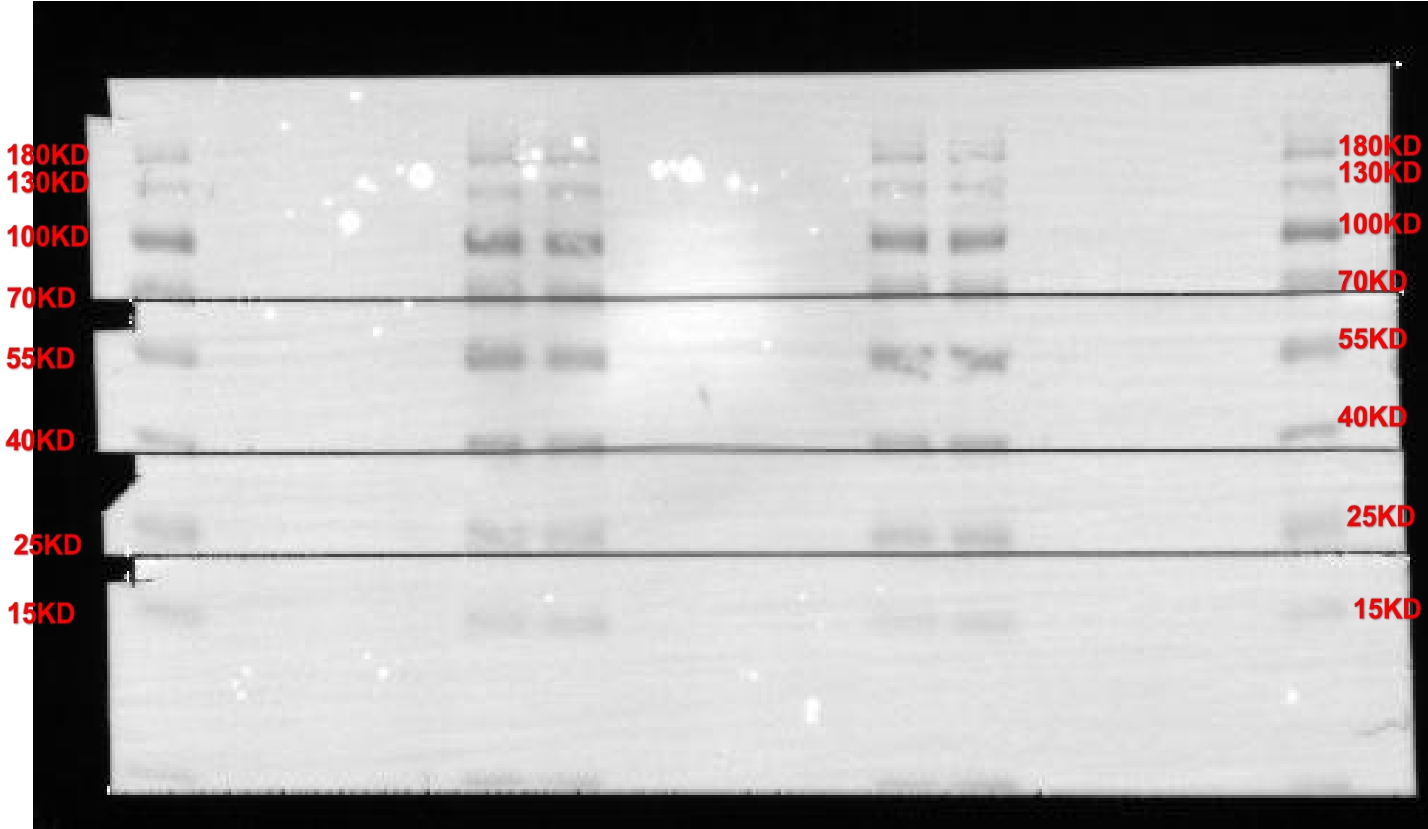

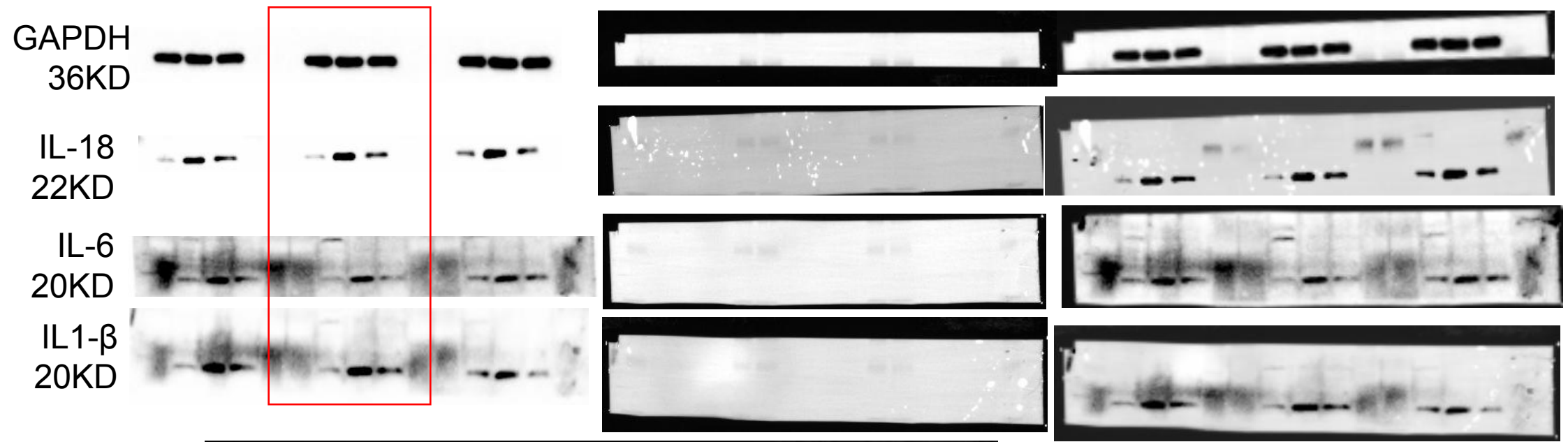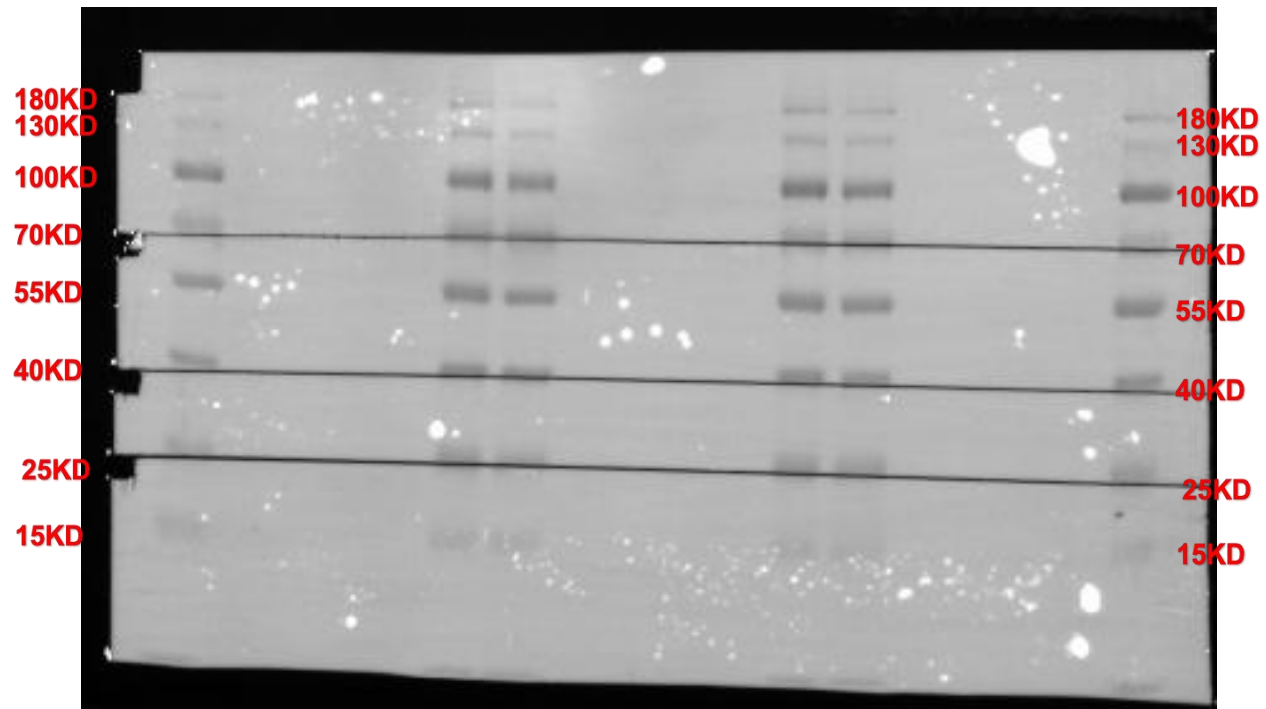

Supplement: Supplementary file 1 [file Datasheet1.pdf]

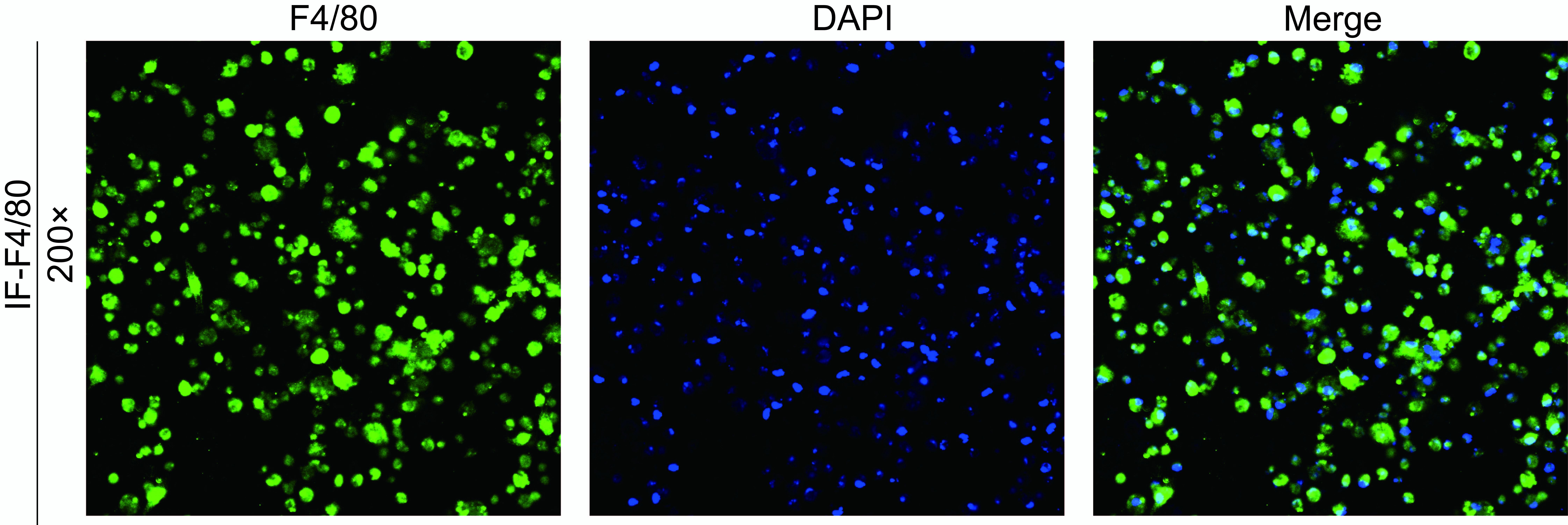

Supplement: Supplementary Figure S1 — Identification of PMs through immunofluorescence. PMs: peritoneal macrophages. [file Image1.jpeg]
